# When health is wealth: occupationally differentiated patterns of health capital in post-industrial Europe

**DOI:** 10.1057/s41285-022-00187-3

**Published:** 2022-10-07

**Authors:** Ivan Harsløf, Kristian Larsen, Clare Bambra

**Affiliations:** 1 Department of Social Work, Child Welfare and Social Policy, Faculty of Social Sciences, Oslo Metropolitan University, Pb. 4, St. Olavs Plass, 0130 Oslo, Norway; 2 Copenhagen University Hospital’s Centre for Health Research (UCSF), Ryesgade 27, 2200 N Copenhagen, Denmark; 3 Institute of Health & Society, Faculty of Medical Sciences, Newcastle University, Royal Victoria Infirmary, Sir James Spence Building, Newcastle upon Tyne, NE1 4LP UK

**Keywords:** Bourdieu, Health capital, Occupational fields, Post-industrial society, Social inequalities in health, Social closure

## Abstract

This paper explores the general relationship between peoples’ health-related practices and their affiliation with different fields in the occupational structure. It argues that ‘healthy behaviour’ may be particularly induced in the field of service occupations (jobs where one is providing a service, rather than producing a physical product), rendering such practices an emerging capital in the sense advanced by Bourdieu. The paper presents an empirical elaboration of this theoretical argument by assessing comparative European data on health behavioural dispositions. Across occupational class levels, defined according to Esping-Andersen’s post-industrial class scheme, service workers display dispositions suggesting greater possessions of health capital than their counterparts in the industrial hierarchy. In a multilevel analysis, considering societal context, the paper furthermore associates such endowments with post-industrial development. Elaborating on the general relationships identified, we suggest the rising importance of individual health investments to be considered as potentially instigating and reinforcing symbolic boundaries (social closure).

## Introduction

Celebrating autonomy, activity, mobility and leanness, and distancing itself from everything dependent, static, immobile and ‘heavy’, a new value system integral to capitalist accumulation has, according to Boltanski and Chiapello (2007), emerged during the late 20th Century. It implies a commodification of areas of life that were previously more shielded from economic forces, including health, appearance and the body and mind (Boltanski and Chiapello 2007, p. 442). This development may partly be driven by the expansion of the service sector, and in broader terms, the unfolding of the post-industrial society (Mears 2014).

Adjusting to the new social order, the importance of peoples’ health-related practices increases. This paper explores these issues by investigating the general associations between peoples’ affiliation with service sector occupations and their health behavioural dispositions. Analogue to other forms of capital (Bourdieu 1984, 1986), health assets that are embodied and displayed through appearance, behaviour and possession of objects signalling an interest in and an ability to nurture a healthy lifestyle, may constitute an emergent social power.

We argue that healthiness may be a more predominant capital in the field of service occupations, with its stimulus towards assets such as appearance and self-presentation and ‘soft skills’ such as flexibility, agility, creativity, collaborative abilities and ‘moral agency’ (Jensen and Prieur 2016).

For health assets to allow agents with such endowments to advance employment opportunities etc., several social mechanisms are likely to play out. A Norwegian study among members of the economic upper class finds that engagement in sport and physical exercise has ‘exchange value’ in the labour market; through recreational sports performances agents successfully establish symbolic boundaries of importance for job recruitment and related processes of inclusion and exclusion (Sølvberg and Jarness 2018). Such inclusion and exclusion may be further reinforced through conversion processes, in which health assets are advancing agents’ acquisition of social capital. The unequal distribution of health assets may ‘naturalise’ social closure; lacking the physical skills and abilities to participate could legitimately exclude certain people from sports related practices, occasions and places where critical social connections are nurtured. Indeed, research shows how people with a negative body image are reluctant to participate in workplace health promotion activities that involve physical exercise (Rossing 2017).

Furthermore, health practices may be stratified by gender. Particularly in post-industrial service-dominated labour markets, ‘women's bodies function as a kind of “sign‐bearing” capital, bestowing status upon organizations and their customers in ways that men's bodies do not’ (Mears 2014, p. 1338).

While much research has examined the relationship between health, lifestyle and levels of education (e.g. Cockerham 2013; Li and Powdthavee 2015; Luy et al. 2019), less focus is directed towards divisions predicated on the horizontal production-services divide. Using data from the Social Inequalities In Health module of the European Social Survey, Round 7 from 2014 (ESS7), this paper explores health behaviour among differently positioned groups in the occupational structure. ESS7 allows us to index health behaviour (nutrition, alcohol/tobacco consumption, physical exercise, etc.), hereby empirically determine the larger theoretical construct of health capital across a wide range of European countries. We use this data to explore ‘general relationships’ for the purposes of theoretical elaboration. Hence, far from being a ‘conformist’ study, it is conducted in the vein of Glaser (2008), to use quantitative data for the flexible generation of theory.

Below, we first discuss the rising interest in healthy lifestyle. In this respect, we discuss some generic features prompting our expectations of different patterns of health-related dispositions in the fields of production and services and their respective hierarchies of occupational class. Second, we present our notion of health capital. Third, we outline the method applied. Fourth, we examine the empirical material, considering individuals both in the contexts of occupational fields, and as nested in larger post-industrial formations. This is followed by a discussion of the results in relation with the general literature. The final section concludes.

## Background

Scholars of the larger societal transformation occurring in the late twentieth century have noted its wide-ranging implications for peoples’ intimate lives. Under the conditions of late modernity, Giddens (1992, p. 31) argues, the body ‘is heavily infused with reflexivity … [to an extent that] it becomes a visible carrier of self-identity and is increasingly integrated into lifestyle decisions which an individual makes’. Bauman (2000, p. 80) points out that this cultivation of the healthy body does not necessarily manifest in ‘asceticism, abstinence or renunciation’. ‘[I]f anything’, he goes on, ‘it means consuming more’. Hence, the middle classes, adapting to the requirements of neo-liberal post-industrial society, are indulging in a growing market for health-related goods and services. These include physical training, mental training, pharmacology, diets, corrective surgery, and a large range of self-monitoring technologies (e.g. Lupton 2016).

Arguably, the health trends affect people not only in their role as consumers, but in all the social roles they assume. Indeed, we can conceive of a structural homology across fields (Bourdieu 1984); labour markets that are increasingly valorising the healthy and fit, encourage workers to consume services and products that bestow upon them these qualities. Workers, in turn, will be attracted by employers offering ‘health promotion’ (e.g. private health insurances, occupational health services, and all sorts of health perks) (Larsen and Harsløf 2021).

Currently, for example, job applicants go to great lengths to let potential employers know about recent accomplishments in sport and exercise (Wallrodt and Thieme 2019; Piopiunik et al. 2020), or downplay or even lie about health conditions in application forms (Riach and Loretto 2009). In some countries, social authorities seek to promote marginalized clients’ employability by offering physical training and diet plans or supporting aesthetic treatments for obesity, crooked and misaligned teeth, and the like (Larsen and Harsløf 2020). Indeed, field experiments have demonstrated that a supposedly unhealthy appearance (e.g. being overweight) is penalized in the job search process. These discriminatory processes apply particularly to female job searchers (Rooth 2009). Even after getting a job, peoples’ appearance may determine further selection into job specific hierarchies and how close one gets to operate vis-à-vis customers and clients (Mears 2014; Boltanski and Chiapello 2007, p. 476n).

Following Bourdieu’s (1984) analyses of the intertwining of the space of lifestyle and social class, we explore the association between health behavioural dispositions and position in the occupational structure. To address this question, we adopt Esping-Andersen’s (1993) post-industrial class scheme. This device is suitable as it describes new divisions of labour and issues of social closure (Leiulfsrud et al. 2005, p. 23). The occupational fields it identifies does not represent independent employment environments (Hertel 2017). Rather, we may think of workers in the two hierarchies as divided by symbolic boundaries. Inside a company made up of production and service occupations, on ‘each step of the vertical hierarchy … autonomy will be larger in post-industrial if compared to Fordist occupations’ (Hertel 2017, p. 89). This is so because the former are ‘characterized by rather blurry authority relations’ (Hertel 2017, p. 87).

Moreover, service occupations stand out by concerning relationships and interactions with outside parties (users, customers, consumers, clients, audiences etc.) (Boltanski and Chiapello 2007, p. 442). Productively engaging in such relations, revolving around sales, presentations, negotiations, conciliations etc., may involve varying degrees of emotional and aesthetic labour (Mears 2014).

Hence, compared with the field of production (let alone primary sector extraction), service work generally implies a high degree of ‘front stage’ exposure (Goffman 1959). Related to this, as Goffman (1959, p. 32) notes, in services there is often a need to ‘dramatize’ one’s acts to demonstrate the value of the product that may otherwise remain imperceptible. In Giddens’ (1990) terms, the task of service workers often lies in re-embedding increasingly abstract relations into personal or recognizable ones.

Consequently, employee characteristics sought by employers have gone beyond traditional skills to include personal attributes and attitudes to the extent that these, as Hampson and Junor (2010) lament, have been included in the modern notion of skills. Furthermore, the increasing obliteration of work and leisure in skilled service sector workplaces (Boltanski and Chiapello 2007, p. 443), even render typical ‘off stage’ activities important, as when sleep becomes ‘the new status symbol’ (*New York Times* 2017).

## Health capital

The partial decline of traditional cultural capital related to ‘highbrow’ consumption has allowed new forms of capital to emerge (Prieur and Savage 2013). Amidst an increasingly dynamic, post-industrial social order, forms of capital that allow agents to display full autonomy, partly supplant the old forms anchored in fixed and static entities, such as property, family bonds and inherited status. The cultivation of ‘leanness’, as in lean organizations and lean manufacturing, and the now ubiquitous call for innovation, illustrates this change (Boltanski and Chiapello 2007). We argue that the strong health trend, accentuating activity and health promotion reflects the emergence of health assets—peoples’ physical condition and healthy appearance and lifestyle—as a new form of capital. Rather than an absence of illness, health is increasingly considered an asset that individuals can optimize (Zinn and Taylor-Gooby 2006, p. 24). This new approach construes the body as an object of investment, encouraging a commodification of health.

The concept of health capital concerns the physical, surgical, nutritional, chemical and mental work around the body (Larsen et al. 2022). It includes the more general exhibition of a self-image as a healthy and active individual, nurtured through more or less conspicuous health consumption and the display of virtuous health-related practices. It is important to stress that we consider such activities as ‘strategies’ and ‘investments’ in Bourdieu’s sense to reflect the largely unconscious relationship between a person’s *habitus* and the field in which the person is embedded (Bourdieu 1993, p. 76).

In contrast to Grossman’s (1972) *substantialist* concept, that considered health capital as an individual’s ‘stock of health’ that can be exhausted, and through medical care, diet, etc., also rejuvenated, a concept building on Bourdieu’s work is *relational.* It posits that effects of capital endowments ‘cannot be reduced to the set of properties individually possessed by a given agent’ (Bourdieu 1986, p. 109). Rather, Bourdieu emphasises the dialectic between agents of different class backgrounds, whereby those with apparently unhealthy lifestyles are in a sense acting as ‘foils’ that by their mere presence accentuate the ‘good’ and ‘moral’ qualities of those with apparently healthy ones (Bourdieu 1984, p. 179). In other words, those failing to display middle-class health behaviours may be further stigmatized and ‘othered’, hereby reinforcing social closure.

There are also structural relations between the socially stratified determinants of health (e.g. material and psychosocial living and working conditions) and the achievement of ‘ideal’ health-related practices—not least due to the cost in expenditure and time of the individual’s accumulation of health capital (Wiggins and Keats 2015).

Our further operationalization of the concept leans on Cockerham, (2009, p. 210), who advises considering peoples’ health practices in terms of *actions or inaction*. Hence, in our empirical assessment, we aim to build a sociological measure of health capital with binary distinctions in various areas of health behaviour.

## The state as a ‘meta-field’

While we argue that health capital may be appreciated differently in production and service occupations, its emergence cannot be isolated to specific occupational fields. Rather, it needs to be considered in the larger societal context. To assess this, we conduct a multilevel analysis. This strategy has been recommended to accommodate the notion of societies as ‘open systems’ (Luke 2004). Indeed, conceptualizations of post-industrial societies emphasize the cross-cutting dynamics across spheres of production and social reproduction, relating to overall accumulation strategies, labour market regulation, patterns of consumption, and family and gender roles. Importantly, it concerns *tendencies* and not something that is ‘already achieved and consolidated’ (Jessop 2018, p. 400). On this background, we determine post-industrialism in terms of the relative presence of four interrelated tendencies: high service sector employment (*tertialization*); a dynamic labour market in terms of job turn-over (*flexibilization*); destabilized family structures (*individualization*) and high proportions of women in the labour force (*feminization*) (Dukes and Streeck 2020; Hertel 2017).

In a Bourdieusian perspective, the mechanisms rendering health an appreciated asset in countries approximating the post-industrial society, not only among people affiliated with the service field, but foisted upon the whole population, can largely be attributed to the state. Bourdieu conceives of the state as a meta-field, regulating through its institutions and instruments of coercion, the various forms of capital, including their ‘exchange rate’ (Bourdieu and Farage 1994, p. 4). Hence, in a service-intensive economy, the state will seek—through all sorts of regulation, investments, educational curricula, and campaigns—to promote individuals’ active participation in service jobs, and, above all, devise systems of classification rewarding those endowed with the relevant skills and mind-sets.

## Method

Using the International Standard Classification of Occupations 2008 (ISCO-08) occupational codes (International Labour Organization 2010), we grouped respondents according to employment sector: primary sector (farming, forestry, mining and fishing), production industries and services. Furthermore, within the two latter sectors (which are the concern of this study), respondents are placed in the occupational hierarchy from unskilled/low-skilled workers to managerial level. The two hierarchies are parallel in terms of skill levels, except that the ‘Professionals and scientists’ group (ISCO skill level IV) in the service hierarchy has no counterpart in the industrial hierarchy.

Table 1 shows the two hierarchies, distinguishing higher and lower positioned groups according to ISCO-88 skill levels.

**Table 1:** Industry and services in the post-industrial class scheme

ISCO-88 skill level	Industrial hierarchy	Post-industrial hierarchy
III–IV	Managers and proprietors (includes executive personnel and the ‘petite bourgeoisie’)	Managers (within service industries)
IV	N/A	Professionals and scientists
III	Clerical, administrative (non-managerial) and sales workers engaged in basic routine tasks of control, distribution and administration	Technicians and semi-professionals (schoolteachers, nurses, laboratory workers, technical designers, etc.)
II	Skilled/crafts manual production workers, including low level ‘technical’ workers	Skilled service workers (cooks, hairdressers, etc.)
I	Unskilled and semi-skilled manual production workers, including transport workers and other manual occupations engaged in manufacture and distribution (packers, lorry drivers, haulers, etc.)	Unskilled service workers or service proletariat (cleaners, waitresses, bartenders, etc.)

*Source* Esping-Andersen (1993) and Leiulfsrud et al. (2005)

We use ESS7e01 (Norwegian Social Science Data Services 2014) which includes data from Austria, Belgium, Czech Republic, Denmark, Estonia, Finland, France, Germany, Ireland, Netherlands, Norway, Poland, Slovenia, Sweden and Switzerland. The post-stratification weights (PSPWGHT) are used in all analyses.

In devising the dependent variable and putting it to work in this study, we lean on Glaser’s (2008) grounded quantitative theory, and in particular, the suggestion to deploy crude indices. This approach rests on the idea of the ‘interchangeability of indices’; underlying phenomena should be detectable through different indicators. Appendix Table 4 lists the variables selected as contributors to the index.

ESS7 included the rotating ‘Social Inequalities in Health’ module, which contains a number of questions on health behaviour (Eikemo et al. 2016). From these questions, we constructed dichotomous variables to reflect seemingly healthy behavioural dispositions in relation to physical activity, intake of fruit and vegetables, smoking, alcohol consumption, and receiving alternative medical treatment (acupuncture, acupressure, Chinese medicine, chiropractic, osteopathy and homeopathy).^1^ The cut-off points of the dichotomous variables are intended to reflect general public health advices on healthy behaviours (Huijts et al. 2017). They were further based on the aim to balance the number of respondents in the categories (obtaining at least 20% in each) to achieve a matrix of binary distinctions.^2^

It is important to stress that this index concerns peoples’ *interest in and ability to nurture a healthy lifestyle,* and not their degree of healthiness. Receiving alternative medical treatment, for example, does not indicate healthiness, but points to an interest in and an ability for self-care (Terry 2002). Aiming to make a *sociological measure*, it is this interest and ability we want to capture.

An exploratory factor analysis (EFA) demonstrated that the six items can be considered as one common factor. Syntax developed by Lorenzo-Seva and Ferrando (2015) was used to obtain the polychoric correlation matrix between the dichotomous variables. The derived correlation estimates provided the input for the EFA. The information on peoples’ intake of fruit and vegetables were strong contributors to the factor, while the remaining items were weaker contributors (see factor loadings in Appendix Table 5). The Kaiser–Meyer–Olkin (KMO) Measure of Sampling Adequacy was 0.583, and hence greater than the 0.50 minimum value required for adequate use of the common factor model (Field 2018, p. 798). The Bartlett's test of sphericity showed that the included variables are correlated (*p* < 0.001). Most of the elements of the anti-image correlation matrix (i.e. a correlation matrix that contains the negatives of the partial correlation coefficients) that are not on the diagonal were small (absolute values ranged from 0.003 to 0.465). Again, this suggests that the model is adequate (Tabachnick and Fidell 2006). The individual KMOs ranged from 0.532 to 0.758, supporting the inclusion of each variable in the factor analysis (Yong and Pearce 2013).

**Table 2:** General linear model

	*B*	SE	Sig.	95% Confidence Interval	
				Lower bound	Upper bound
Intercept	3.503	0.101	***	3.305	3,702
Male (female = ref)	− 0.598	0.016	***	− 0.630	− 0,566
Age	− 0.018	0.002	***	− 0.022	− 0,013
Years of full-time education completed	0.020	0.003	***	0.015	0,026
No 3 months history of unemployment (unemployment history = ref.)	− 0.219	0.018	***	− 0.253	− 0,184
No migration background (migration background = ref.)	− 0.178	0.023	***	− 0.222	− 0,133
Occupational field (professionals and scientists = ref.)					
Primary sector	− 0.059	0.048	n.s	− 0.153	0,036
Managers in industrial production	− 0.121	0.043	**	− 0.205	− 0,037
Clerical. administrative and sales workers	− 0.115	0.031	***	− 0.175	− 0,055
Skilled/crafts manual production workers	− 0.248	0.035	***	− 0.317	− 0,178
Unskilled/semi-skilled manual production workers	− 0.265	0.038	***	− 0.339	− 0,190
Managers in service production	− 0.072	0.038	n.s	-0.146	0,002
Technicians/semi-professional service workers	0.062	0.031	*	0.001	0,123
Skilled service workers	0.019	0.043	n.s	− 0.066	0,104
Unskilled service workers	− 0.119	0.035	***	− 0.187	− 0,051
Missing data on occupational variable	− 0.054	0.039	n.s	− 0.130	0,022
Self-rated health (‘Very bad = ref.’)					
‘Very good’	0.595	0.079	***	0.441	0,749
‘Good’	0.417	0.078	***	0.265	0,569
‘Fair’	0.353	0.078	***	0.201	0,506
‘Bad’	0.280	0.083	***	0.118	0,443
Country of residence (Slovenia = ref.)	0^a^				
Austria	− 0.250	0.047	***	− 0.341	− 0,158
Belgium	− 0.327	0.047	***	− 0.419	− 0,235
Switzerland	0.076	0.049	n.s	− 0.020	0,172
Czech Republic	− 0.358	0.047	***	− 0.450	− 0,265
Germany	0.022	0.043	n.s	− 0.062	0,106
Denmark	− 0.103	0.049	*	− 0.198	− 0,007
Estonia	0.132	0.046	**	0.042	0,222
Finland	0.400	0.046	***	0.311	0,490
France	− 0.107	0.046	*	− 0.197	− 0,016
Ireland	0.084	0.046	n.s	− 0.006	0,175
Netherlands	− 0.464	0.045	***	− 0.551	− 0,376
Norway	− 0.010	0.049	n.s	− 0.106	0,087
Poland	− 0.219	0.048	***	− 0.312	− 0,125
Sweden	0.043	0.047	n.s	− 0.050	0,135
*R*^2^ = 0.118					
*n* = 27.901					

Dependent variable: health capital index

The results presented below are derived from a general linear model. As the dependent variable, we use the raw scores on the index, with high scores indicating behavioural dispositions that reflect interest and ability to invest in one’s health. According to DiStefano et al. (2009), this choice is most desirable when dealing with a scale that has been constructed in an exploratory fashion (with secondary data). However, to test the robustness of the analysis, models with alternative methods of computing the dependent variable have also been run using weighted summed scores (created by multiplying the factor loading of each item by the raw score for each item), and regression-based factor scores. Using these alternative constructs of the dependent variables did not alter the association with the occupational variable.

Even if occupational hierarchies reflect skill levels, we considered schooling with a variable on years of full-time education completed. This allows us to account for ‘over-education’, i.e. individuals with a higher level of education than required by their current/most recent jobs.^3^ We further included information on previous unemployment, the respondents’ self-reported health, and country of residence.

To explore the salience of the larger post-industrial context beyond individual’s placement in the occupational hierarchy, as an additional step, we conducted a multi-level analysis. As a country level variable measuring post-industrial development, we combined four standardized indicators (see Appendix Table 6):

- *Tertiarization:* Proportion of service jobs
- *Flexibilization:* Incidence of job tenure, less than 12 months
- *Individualization:* Proportion of single-adult households
- *Feminization of labour markets:* Gap between female and male employment rate (reversed)

**Table 3:** Multi-level analysis—dependent variable: health capital index

	Model 0			Model 1			Model 2		
	*B*	SE	Sig.	*B*	SE	Sig.	*B*	SE	Sig.
Fixed part									
Constant	3.158	0.056	***	2.517	0.085	***	2.516	0.081	***
Gender (0 = male; 1 = female)				0.625	0.016	***	0.625	0.016	***
Age				− 0.002	0.000	***	− 0.002	0.000	***
Migration background (0 = no migration background; 1 = has migration background)				0.185	0.022	***	0.185	0.022	***
Previous unemployment (0 = has been unemployed for 3 months; 1 = no 3 months unemployment history)				0.231	0.017	***	0.231	0.017	***
Self-rated health (‘Very good’ = Ref.)									
‘Good’				− 0.188	0.019	***	− 0.188	0.019	***
‘Fair’				− 0.259	0.023	***	− 0.258	0.023	***
‘Bad’				− 0.331	0.038	***	− 0.331	0.038	***
‘Very bad’				− 0.631	0.076	***	− 0.631	0.076	***
Years full-time education completed				0.019	0.003	***	0.019	0.003	***
Occupational field (Professionals and scientists = Ref.)									
Missing information on occupation				− 0.002	0.038	n.s	0.000	0.038	n.s
Primary sector				− 0.050	0.048	n.s	− 0.050	0.048	n.s
Managers in industrial production				− 0.119	0.042	**	− 0.118	0.042	**
Clerical. administrative and sales workers				− 0.107	0.030	***	− 0.107	0.030	***
Skilled/crafts manual production workers				− 0.234	0.035	***	− 0.233	0.035	***
Unskilled/semi-skilled manual production workers				− 0.264	0.038	***	− 0.262	0.038	***
Managers in service production				− 0.071	0.038	n.s	− 0.071	0.038	n.s
Technicians/semi-professional service workers				0.059	0.031	n.s	0.059	0.031	n.s
Skilled service workers				0.029	0.043	n.s	0.030	0.043	n.s
Unskilled service workers				− 0.116	0.034	***	− 0.114	0.034	***
Measure of post-industrial development							0.134	0.062	*
Interaction term: Post-industrial development × gender							− 0.019	0.019	n.s
Random part									
*σ*_u_^2^ (country-level variance)	0.047	0.017		0.046	0.017		0.036	0.014	
* σ*_e_^2^ (individual-level variance)	1.717	0.014		1.555	0.013		1.555	0.013	
Model fit									
− 2 × log-likelihood		94,939.96			91,551.61			91,546.97	

***p < 0.001; ** p < 0.01; * p <  0.05

*SE*  standard error. *n*_*i*_ = 27.901: *n*_*j*_ = 15

## Results

The general linear model (Table 2) demonstrates the following associations between the independent variables and the health capital index. Younger people tend to score higher on the index. Females have a higher index score than males. People born in a foreign country or with both parents from a foreign country have higher scores than people who do not. Regarding unemployment history, people who have been unemployed and seeking work for a period of more than three months have lower index scores than others. As can be expected, the index is positively correlated with schooling as measured in years of completed full-time education, and with self-reported health.

**Fig. 1:**
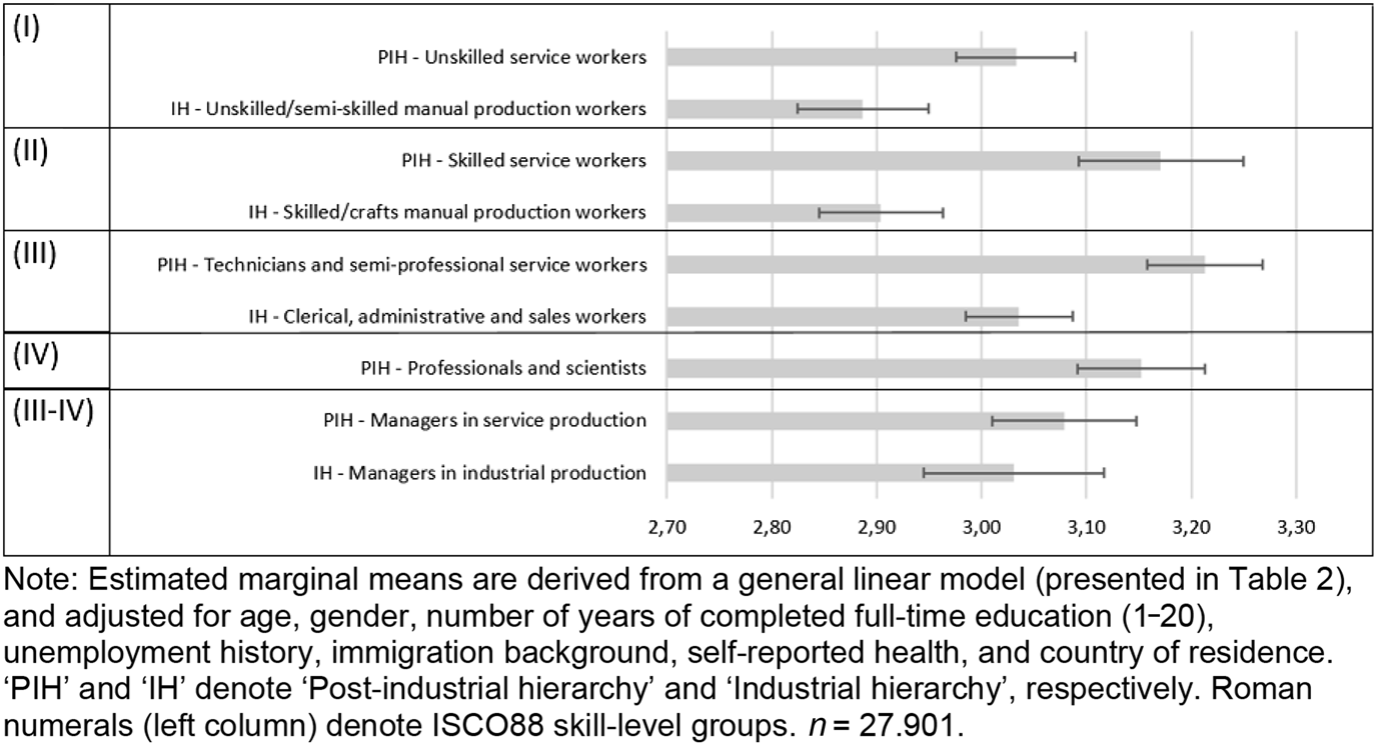
Estimated marginal means of Health behaviour index by position in industrial and post-industrial hierarchies (with 95% confidence intervals)

With these factors being adjusted for, the model suggests some interesting associations between affiliations within occupational hierarchies and scores on the health capital index. Figure 1 depicts the estimated marginal means on the index for the different skill-level groups across the industrial and post-industrial hierarchies.

We observe that at skill level II, service workers score slightly higher on the index than their counterparts in the industrial hierarchy. Similarly, at level III, technicians and semi-professionals in the post-industrial hierarchy score higher than the clerical, administrative and sales personnel in the industrial hierarchy. Estimated index means for the post-industrial managerial and unskilled worker levels are also higher than those of their respective counterparts in the industrial sector. It is only at the management level that we find no significant difference that can be attributed to affiliation with the industrial and post-industrial fields.

We next consider if residing in a country characterized by advanced post-industrial development has an independent effect. The underlying idea is that mechanisms inducing individual health investments cannot be isolated to a specific location in the social space (one’s affiliation to an occupational field), but is also affected by overarching forces, most prominently the state as a ‘meta-field’.

Table 3 presents the results of a stepwise multilevel analysis, with an empty model, a model with individual variables, and a full model that also includes contextual variables. The analysis demonstrates that higher individual health capital is indeed associated with an advanced state of post-industrial development. We observe that the context variables account for 22% of the explained variance in the full model. Using a cross-level interaction term, the model further explores if the context of advanced post-industrial development induces extra pressure on women when it comes to health behaviour. However, that argument is not corroborated.

## Discussion

Overall, the tendencies found in the analyses align well with the general literature. Indeed, research suggest that notably younger cohorts have adapted to the health trend (Goodyear et al. 2019). The rather large difference between women and men is consistent with studies on gender differences in healthy lifestyle, which consider other indicators (ranging from women more frequently visiting a doctor for preventive care to using seatbelts when driving; Cockerham 2013). The tendency of people of migrant background showing higher index scores may be explained with reference to so-called ‘cultural buffering’, a notion adopted to understand the ‘healthy migrant effect’ in a European context (Alexander et al. 2012).

As regards the differences across occupational fields that we are interested in, we notice that our findings resonate with the literature, although we do not find studies on health behaviour using the post-industrial class scheme, as done in the present study. Using a rough distinction between industrial and office work, in an analysis restricted to middle-aged Finnish men, Näslindh-Ylispangar et al. (2008) found that industrial workers were less motivated for changing unhealthy behaviours. A Norwegian qualitative study (Gjernes 2013), also among male industrial workers, showed how taking care of one’s health, for example avoiding certain work-tasks when recovering from an injury, was difficult due to peer pressure and management’s lack of concern. Highlighting such dynamics within peer groups and organizational contexts is important to understand the occupationally differentiated patterns that we find.

## Conclusion

Our exploration of the comparative data suggests that across individuals residing in different European countries it is possible to consider a number of health behavioural dispositions under one unifying factor. We do not regard this factor as reflecting degrees of health in a substantial matter. Rather, as a sociological concept, we take high scores to indicate awareness of lifestyle choices generally considered conducive to personal health, implying an interest in and ability for self-care. We have proposed this health behaviour as (a part of) an emergent form of capital, in a Bourdieusian sense, as societies enter a post-industrial social order.

With this crude index, we have found some general relationships indicating that inequalities in health behaviour not only span the vertical occupational hierarchy as traditionally defined by skill level and managerial authority but are also associated with peoples’ sectoral field-affiliations. Hence, people employed in the expanding service sector appear better endowed with assets and inclinations subsumed under the health capital concept.

Of course, such general relationships in no way imply one-way causality, as if entering a specific occupational group somehow triggered a healthy lifestyle. Rather, we should understand occupational strata to represent broader ‘contexts’ (Pawson 2000), or Bourdieusian fields, i.e. arenas for the production, circulation and appropriation of material and immaterial resources. Thus, we wish to move the emphasis from individual behaviour—or ‘lifestyle choices’—to the social and material conditions that shape health behavioural dispositions. This approach recognizes how a specific occupational field is homologously structured by adjacent fields (Wang 2016). In that respect, we may consider how a ‘taste for health’ may already be stimulated in the educational field as individual agents prepare themselves for a career in service occupations (cf. Larsen et al. 2022).

That said, the service sector may well constitute a context that also actively induces interest in health and self-care. We know for example that occupational health promotion programmes have a much stronger footing in service sector workplaces than in manufacturing (O’Donnell 2002). Moreover, processes of selection into and exclusion from occupational fields, and discriminatory processes within them, may determine the possession of health capital characterizing the agents occupying them.

Hence, we suggest that existing approaches considering health assets as a social power in the Bourdieusian sense (e.g. Shim 2010; Collyer et al. 2015) expand the perspective beyond patients’ interaction with health providers.

Our analysis suggests that people in countries dominated by features of post-industrial development are more likely to invest in their own health. As societies move towards post-industrial conditions, perhaps people generally become more aware of how to maintain a healthy lifestyle. Even if unevenly distributed, this awareness, it could seem, eventually permeates across different strata of society. Nevertheless, by adopting Bourdieu’s notion of capital, we have taken an agonistic perspective on the role of health assets in post-industrial society. Considered as a form of capital, health endowments should be perceived as relative. From such a perspective, even small differences may be salient in determining differences in status and access to resources and positions (e.g. career opportunities). Individual health investments may be celebrated by the state and elite groups, because ‘dominant classes are only dominant if they successfully impose their sort of capital as the dominant principle of hierarchisation’ (Vandenberghe 1999, p. 53n).

There can be important societal implications of such processes of health assets being leveraged for purposes of domination. Some social groups’ overt investments in health may alienate and displace others from doing so. This may induce a polarization in health behaviour. In some countries the COVID-19 crisis showed how some social groups’ embracing of infection protection behaviour induced other groups’ overt neglect (*The Guardian* 2020–06–29). Policy makers wanting to alleviate social inequalities in health should be aware of how health behaviour increasingly functions as a social marker in wider systems of oppositions, also beyond the production-services divide that has been the focus of the present study.

A limitation of the adopted perspective is its focus on an individual dimension of health assets. Arguably, collective resources such as access to free or subsidized health services could be factored into a broader notion of health capital. New research is needed to explore if such collective resources are relieving individuals from the need for personal bodily investments, in the manner previous research has studied the impact of the welfare state on individuals’ relationship to the labour market and family.

In the context of growing emphasis on healthy lifestyle, disposing of health assets are likely to become a social power, and people with relatively few such assets may increasingly interpret their marginality as caused by their own bad health choices. Indeed, research has shown how people of lower social classes, particularly women, tend to dislike their own physique (Vandebroeck 2015).

Moreover, narratives accompanying the deep-seated health trend tend to emphasize individual agency. Hereby the congenital physique and the hereditary nature of some diseases and disorders to which people are prone is downplayed. The trend is furthermore ignoring the social determinants of health. Across Europe, ample evidence exists on the interlinkage of health and social status. This concerns inequalities in living conditions affecting health such as quality of nutrition, access to recreational facilities and exposure to pollution, as well as inequalities in treatment, such as higher income groups being able to bypass waiting lists (Fjær et al. 2017).

The individualized narrative may lead people to attribute marginal social positions to poor lifestyle choices. This in turn may legitimate the growth of social inequalities and undermine collective efforts towards social security, welfare and public health provisions.

## Appendix

See Tables 4, 5 and 6.

**Table 4:** Frequency table of the contributors to the health behaviour additive index

Variable	*N*	%
*Little alcohol* Does not drink alcohol several times a week/every day		
No	6414	22.7
Yes	21,705	76.9
Missing	102	0.4
*Fruit* Eats fruit every day		
No	8972	31.8
Yes	19,240	68.2
Missing	9	0.0
*Vegetables* Eats vegetables at least twice a day		
No	21,596	76.5
Yes	6612	23.4
Missing	13	0.0
*Physically active* Is physically active for 30 min (in total) at least 4 days per week		
No	6688	43.8
Yes	15,825	56.1
Missing	24	0.1
*Alternative medicine* Received some kind of alternative medical treatment in past 12 months		
No	17,439	61.8
Yes	10,782	38.2
Missing	0	0
*No smoke* Does not smoke		
No	13,227	46.9
Yes	14,974	53.1
Missing	20	0.0

*N* = 28,221

**Table 5:** Factor loadings: health behaviour index

	Factor 1
Little alcohol	0.128
Fruit	0.767
Vegetables	0.630
Physically active	0.235
Alternative medicine	0.171
Not smoking	0.250

**Table 6:** Indicators of post-industrialism by the year the ESS7 survey was conducted

	Incidence of job tenure, less than 12 months, 2014^a^	Proportion of single-adult households, 2014%^b^	Proportion of employees working in services, 2014%^c^	Gaps between male and female employment rate, 2014, percentage points^d^
Austria	14.6	40	77	10.3
Belgium	11.4	36	69	9.6
Czech Republic	10	37	59	17.6
Denmark	21.9	52	78	8.1
Estonia	15.7	52	66	8.8
Finland	17.8	45	74	3.6
France	12.5	42	77	8.2
Germany	13.4	45	71	10.6
Ireland	14.4	34	77	11.1
Netherlands	14.4	42	83	11.6
Norway	14.9	43	77	4.9
Poland	12.1	28	58	15.4
Slovenia	9.3	39	60	11
Sweden	19.5	63	80	4.8
Switzerland	16.1	36	76	11.9

^a^https://www.oecd-ilibrary.org/docserver/empl_outlook-2016-en.pdf?expires=1602061412&id=id&accname=oid023401&checksum=ADBE32458B97D6A3C918044DF6C41C8C

^b^https://appsso.eurostat.ec.europa.eu/nui/submitViewTableAction.do

Norway (2015): https://www.ssb.no/befolkning/statistikker/familie/aar/2016-10-28; Switzerland (2018): https://www.bfs.admin.ch/bfs/en/home/statistics/population/effectif-change/households.html

^c^Employed in services (ISIC rev.4, G-U) as a proportion of all employees (ISIC rev.4, A-U). *Source* Eurostat 2020 (https://stats.oecd.org/Index.aspx?DataSetCode=ALFS_EMP)

^d^https://stats.oecd.org/index.aspx?queryid=54742#

## Footnotes

1 An initial analysis also included a variable on time spent watching television (to reflect sedentary lifestyle). However, in the EFA, this item had an individual KMO-MSA on only 0.513 in the Anti-image correlation matrix. While it is just above the 0.5 threshold recommended when determining a factor, the substantial argument that many people have partly replaced television with alternative platforms (smartphones etc.), made us omit it. Doing this slightly improved the performance of the EFA (from 25.705 to 29.054% of total variance explained). Removing television watching from the index, on the other hand, did not change the general relationships with occupational affiliation.

2 As an alternative way of constructing the index, we also standardized the six variables before combining them in an additive index. This procedure utilizes more of the information contained in those of the variables with a range of answer categories. Also with this alternative measure of health capital as dependent variable we find significant differences between respondents affiliated with the different strata of the the production and service fields.

3 Research has found that it is within the service sector that over-education is simultaneously being the most prevalent (in the administrative and hospitality sectors) and the least prevalent (in the health and education sectors) (Tarvid 2015).
